# grafify online: A web app for graphing and statistical analysis

**DOI:** 10.1371/journal.pbio.3003909

**Published:** 2026-08-03

**Authors:** Avinash R. Shenoy

**Affiliations:** Department of Infectious Disease, Imperial College London, London, United Kingdom

## Abstract

Creating accurate graphs and performing statistical analyses can be challenging for those who lack coding experience. This Community Page introduces grafify online, a free, browser-based app for generating publication-quality graphs and performing statistical tests and post-hoc comparisons.

## Teaser

grafify online is a free, browser-based app for generating publication-quality graphs and performing statistical tests and post-hoc comparisons. Users can customize plots and explore statistical results without needing R programming experience.

## Introduction

Data visualization is a cornerstone of the scientific enterprise. Simple and clear graphs help scientists identify patterns and draw conclusions. Similarly, formal statistical analyses are central to data interpretation and science communication, especially in publications and presentations. However, creating accurate and aesthetically pleasing figures and performing statistical analyses can be challenging for users with no coding experience and without access to commercial software. While online apps for data visualization are available [1–3], opportunities for statistical analysis are currently limited. grafify online bridges this gap by providing a guided web-app for general purpose data plotting and statistical analyses without needing coding experience.

Despite being open source, wider use of the R programming language by scientists is limited, probably due to the learning curve associated with packages required for plotting (e.g., ggplot2 [4]) and statistical analyses (e.g., lme4 [5], emmeans [6]). Therefore, grafify was developed as an ‘all-in-one’ R package for graphs and statistics, and as an open-source tool for teaching biostatistics and R coding to students [7].

## Motivation and user interface

A key goal while developing grafify online was to link visual data interpretation with statistical analysis, ensuring that ‘eyeballing’ data is accompanied with formal analysis. grafify online offers linear models as modern alternatives to ANOVAs, including more advanced mixed models, for null-hypothesis significance testing (NHST). These are better suited for randomized block designs and experiments with repeated measures, which are common in biology, but scientists often lack accessible tools to analyze them.

**Fig 1:**
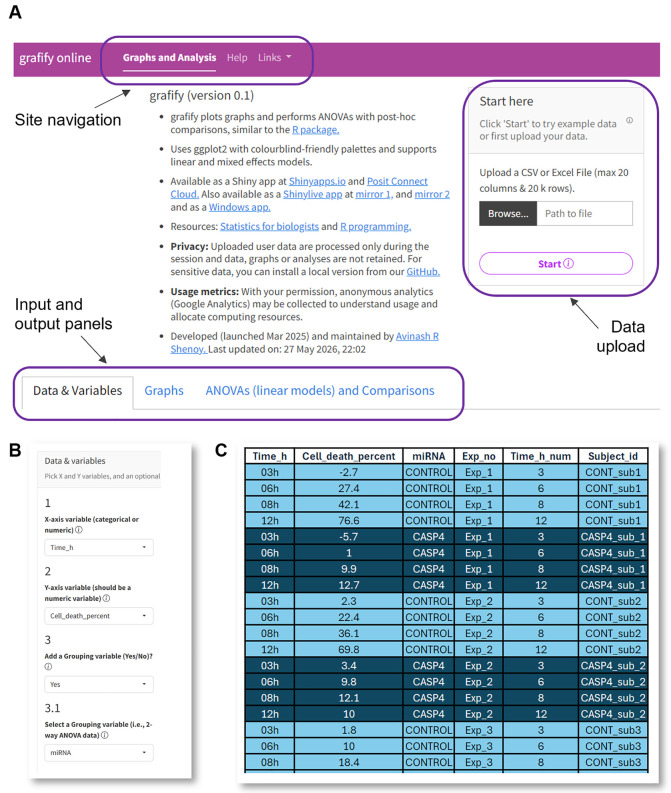
The user interface and data format for grafify online. **A.** The grafify online landing page provides help and navigation on top, start panel on right and tabs below for user inputs and app outputs. **B.** Example of Boxes that guide users, showing the example data set available in grafify online. Boxes 1–3.1 are for choosing variables from user data for plotting graphs and analysis. At least one variable for X- and Y-axes should be chosen in Boxes 1 and 2. Box 3 and 3.1 are optional for two-way ANOVA designs. **C.** Part of the example dataset in grafify online is shown. User data should be in the long or ‘tidy’ data table format, which has one variable per column and unique values across rows.

The web app plots and analyzes one-way or two-way ANOVA designs using linear and mixed effects models. The grafify online interface is organized into three panels or tabs, guided by ‘Readme’ instructions, helpful tooltips, and an example dataset with randomized block and repeated measures (Fig 1A). The three panels are: ‘Data and Variables’, for data upload and choosing variables for graphs and analyses; ‘Graphs’, for plotting, customizing, and downloading graphs; and ‘ANOVAs (linear models) and Comparisons’, for linear model fitting, diagnosis of model residuals, post-hoc comparisons, and effect sizes.

Numbered boxes guide users through the steps (Boxes 1–10 online; Boxes 1–3 are shown in Fig 1B). Variables chosen for the X- and Y-axes of the graph (i.e., the fixed factor(s) and the response variable, respectively) are also used in statistical analyses. Data containing common exploratory variables, including categorical (e.g., treatment, genotype, sex) and numeric (e.g., time, temperature, concentration) variables can be plotted and analyzed. Users choose statistical tests based on their experimental design; therefore, best practice defaults are applied only for visualization and not for statistical tests. Detailed usage instructions are available on the online ‘Help’ page, and a brief overview of the approach and features is provided below.

### Data input format and variables

grafify online expects data in a ‘tidy’ (long) format as CSV or Excel files (with no spaces in column names; Fig 1C). A drop-down list of variables from the user’s data table allows the selection of X- and Y-axis groups for graphs and analyses. An optional grouping variable can be chosen for two-way ANOVA designs.

### Graph types

**Fig 2:**
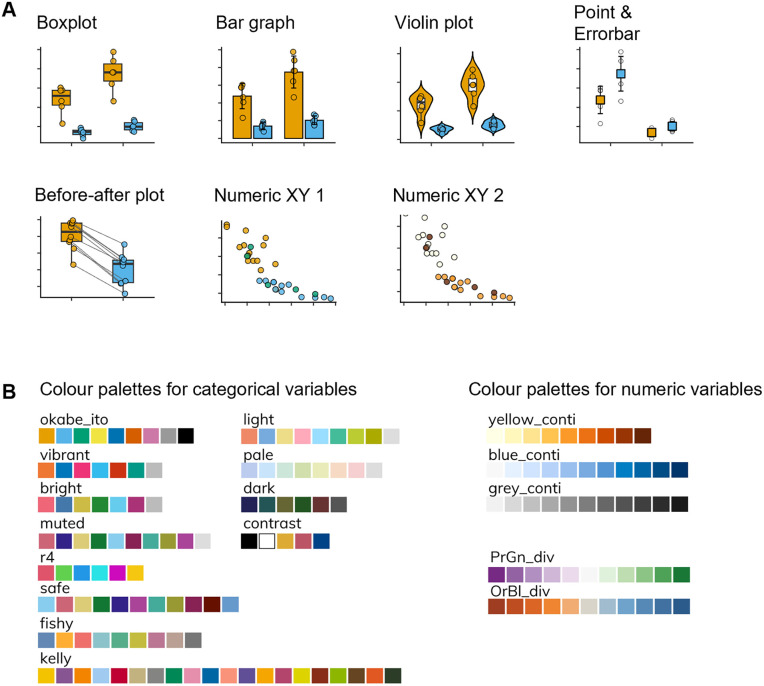
Types of graphs and color schemes available in grafify online. **A.** Examples of graphs that can be plotted with grafify online. Box and whiskers and violin plots show a line at the median with a box representing the interquartile range (IQR) and whiskers that show 1.5× IQR. Bar graphs and point and error-bar plots show the mean, and the dispersion can be shown as standard deviation (default), standard error of mean, or 95% confidence intervals. Before–after plots show matched data. Numeric XY graphs have numeric variables on both and X- and Y-axes and require a grouping variable that is either categorical (Numeric XY 1) or numeric (Numeric XY 2). **B.** Categorical (left) and numeric (right) color palettes that are accessible to colorblind individuals.

grafify online offers a variety of plot types based on user-selected variables (e.g., categorical or numeric) [4,7]. For transparent reporting, all data points are displayed along with the central tendency (mean or median and interquartile range [IQR]) and dispersion (1.5× IQR, standard deviation, standard error of mean, or 95% confidence interval calculated using the *t*-distribution; Fig 2A). Additional graph types include before–after plots for matched data and data distribution histograms/density plots.

### Customizing and downloading graphs

The appearance of graphs can be customized extensively (see details online). A key feature is the Okabe-Ito color palette as default, with options to choose from among 17 palettes that are accessible to colorblind individuals (Fig 2B), or to assign user-preferred colors. Data symbols can be customized by changing their shape, size, opacity, and colors. Graph axes can also be changed from linear (default) to logarithm (base 10 or 2) scales, as log scales are useful for many biological data (e.g., bacterial survival data, viral titers, qRT-PCR). If axes use a log-transformation, statistical tests will apply it, too. This is because plotting on a log-axis suggests a log-normal distribution of raw data and potentially a normal distribution of residuals of linear models (ANOVAs) fitted to log-transformed data. Graphs can be downloaded as PDF files, which are vector graphics suitable for publication.

### Statistical analyses

After plotting a graph, users can choose to fit a simple or mixed linear model, visualize the distribution of residuals (i.e., perform model diagnostics visually), and obtain the ANOVA table calculated from the fitted model. Linear models for analysis of biological data have been discussed before [8,9], and only a brief overview is provided below.

#### Linear models.

In the simplest case of comparing two groups with a Student *t* test, NHST involves fitting an ordinary least squares (OLS) line through data (a linear model) and asking whether this line has a significantly non-zero slope. The null hypothesis is that the means of the two groups are the same (i.e., the slope of the line is zero). As with traditional tests, a key requirement (in addition to the independence of observations and random sampling) is that the residuals of the OLS fit are approximately normally distributed.

Heavily skewed residuals of linear models could indicate that the model is not a good fit for the data and that the statistical results may be unreliable. Therefore, grafify online helps users assess model fit by plotting the distribution of residuals as a quantile–quantile (QQ) plot and as a residual density distribution plot. If the residuals appear skewed, the linear model may not be a good fit for the observed data and NHST conclusions may be unreliable. A potential solution is to transform the data (e.g., log-transformation, as discussed above, or others).

#### Mixed models.

Mixed models are used to analyze experimental designs that have ‘mixed’ factors, i.e., that contain fixed effects (factors that are of interest) and random effects (those that are not of interest). Fixed effects are of scientific interest because we want to know whether they affect the response variable; examples include treatment, genotype, sex, time, and concentration. By contrast, random factors affect the outcome variable and contribute to noise (standard error) that we want to account for, but we are not interested in comparing them per se. Examples include subjects measured repeatedly over time or experimental repeats (block designs) (Fig 1C) [10].

*P* values derived from linear models require accurate degrees of freedom, which should reflect statistically independent (i.e., biologically independent) observations [9,10]. Overestimation of degrees of freedom results in inaccurately low *P* values and false positives. To avoid this, grafify online provides an option to calculate the mean of technical replicates within levels of the random factor before model fitting. Users must average technical replicates before using simple linear models.

Various post-hoc comparisons are possible if the linear model fit is acceptable. Estimated marginal means from the fitted model and two-tailed *P* values for comparisons, their effect sizes, and 95% confidence intervals are provided [6]. By default, *P* values are corrected for multiple comparisons using the Benjamini and Hochberg false-discovery rate method (with *Q* = 0.05).

### User data handling

In the app hosted on shinyapps.io or Posit Cloud Connect, the uploaded data file persists temporarily on the server within the user’s session and is lost upon inactivity or at the end of the active session; user data are not accessible by developers. By contrast, Shinylive uses WebAssembly technology through webR for serverless execution of R code [11] (available from https://grafifyonline1.shenoylab.com/ and https://grafifyonline2.shenoylab.com/). Therefore, the ‘uploaded’ user data file and analyses remain local within the user’s browser [11]. In both apps, optional anonymous user metrics are collected with permission to measure usage and allocate computing resources. In the Windows standalone app, analysis is off-line, data remain local to the user, and no usage metrics are collected. For sensitive data, users are encouraged to install a local Windows copy or the Shiny app from the GitHub repository (also available at Zenodo: DOI: 10.5281/zenodo.21264255).

## Conclusions

The grafify online app fills a key gap by enabling mixed effects analysis, post-hoc comparisons, and effect sizes in a browser, as well as the creation of free, high-quality illustrations. Additionally, grafify online is a useful educational tool to introduce linear models and mixed effects to students and early career researchers. A limitation is the that currently only up to two fixed factors and up to two random factors are fit using random intercepts models; however, interested users can use the grafify R package for complex experimental designs.

## Abbreviations

IQR: interquartile range
NHST: null-hypothesis significance testing
OLS: ordinary least squares
QQ: quantile–quantile
